# Responding to the Spiritual Needs of Palliative Care Patients: A Randomized Controlled Trial to Test the Effectiveness of the Kibo Therapeutic Interview

**DOI:** 10.3389/fpsyg.2020.01979

**Published:** 2020-08-21

**Authors:** Ana Soto-Rubio, Marian Perez-Marin, David Rudilla, Laura Galiana, Amparo Oliver, Miguel Fombuena, Pilar Barreto

**Affiliations:** ^1^Department of Personality, Evaluation and Psychological Treatment, Faculty of Psychology, University of Valencia, Valencia, Spain; ^2^Air Liquide Healthcare, Valencia, Spain; ^3^Department of Methodology of Experimental and Social Sciences, University of Valencia, Valencia, Spain; ^4^Dr. Moliner Hospital, Valencia, Spain

**Keywords:** palliative care, spirituality, intervention, treatment effectiveness, psychological resilience

## Abstract

**Context:**

The WHO recognizes the need to attend to patients’ spiritual needs as being fundamental to comprehensive and high-quality end-of-life care. Spiritual needs must be attended to since the resolution of biological and psychosocial issues is insufficient to reduce patients’ suffering. Associations have been found between spiritual needs and other variables of importance for patients in palliative care. Despite the consensus that exists regarding the importance of assessing and attending to spiritual needs, professionals encounter many difficulties in attempting to do so.

**Objectives:**

Our study aims to demonstrate the benefits that the Kibo therapeutic interview in palliative care patients can have for spirituality, demoralization, and resilience.

**Methods:**

A parallel randomized controlled trial of two groups was undertaken. Information on 60 palliative care patients during pre- and post-intervention time points was gathered.

**Results:**

ANOVAs showed a statistically significant effect of the intervention on the dimension of transpersonal spirituality. The ANCOVA for the effect of the intervention on resilience also pointed to its effectiveness. When the means of demoralization were examined, a higher decrease in the levels of demoralization for patients in the intervention group was observed, when compared to patients in the control group.

**Conclusion:**

Our findings point to this interview as an effective means to attend to the spiritual needs of palliative patients, reducing demoralization and increasing resilience. Future research could focus on a broader sample and on the effects of this interview on family caregivers, mourners, and health care professionals.

**Clinical Trial Registration Number:**

https://clinicaltrials.gov/ct2/show/ Identifier NCT03995095.

## Introduction

Spirituality is seen as an inherent and idiosyncratic characteristic common to all human beings (Tanyi, 2002; Bartlett et al., 2003; dos Santos et al., 2018) and as a concept that is more holistic and inclusive than that of religion, which it both includes and transcends (Macrae, 1995; Tanyi, 2002; Sinclair et al., 2006; Vachon et al., 2009; Visser et al., 2010). The WHO recognizes the need to attend to patients’ spiritual needs as being fundamental to comprehensive and high-quality end-of-life care (World Health Organization, 2002). The need to take a holistic approach to patient needs has been affirmed by a wide range of researchers (Baines and Norlander, 2000; Chochinov, 2002; Draper, 2012; Otis-Green et al., 2012; Puchalski, 2012). In particular, at the end of life, issues related to spiritual needs emerge: the need to find meaning and coherence, to feel connected to a greater reality, and to transcend (Benito et al., 2014). By addressing spiritual needs effectively, health care professionals can help the patient at the end of his/her life find meaning, maintain hope, and, to some extent, accept death (Corr and Corr, 2012).

Associations have been found between spiritual needs and a range of variables of importance for patients in palliative care (Fombuena et al., 2015), such as quality of life (Rajmohan and Kumar, 2013; Haugan, 2014) and physical health (Koenig et al., 2004; Delgado-Guay et al., 2011; Hui et al., 2011; Kandasamy et al., 2011; Haugan, 2014; Fombuena et al., 2015).

In the field of end-of-life care, research has found associations between meeting spiritual needs and the following: higher affective well-being in general (McClain et al., 2003; Breitbart, 2007; Krikorian and Limonero, 2012; Powers et al., 2018), fewer desires for hastened death (McClain et al., 2003), greater emotional well-being (Koenig et al., 2004; Haugan, 2014), greater resilience and effective symptomatic control (Fombuena et al., 2015), and fewer depression symptoms and concerns about death (Edmondson et al., 2008).

Despite the consensus that exists regarding the importance of assessing and attending to spiritual needs, professionals encounter many difficulties in attempting to do so (Chochinov and Cann, 2006; Selman et al., 2011; Galiana et al., 2013). Despite these intrinsic difficulties, the assessment of spirituality is assumed to be a challenge that health care staff must face (Miller and Thoresen, 2003).

The Spirituality Research Group (GES) of the Spanish Society for Palliative Care (SECPAL) has developed a comprehensive and transconfessional model (Benito et al., 2014) in which spirituality is understood to be:

“our essential nature, from which emerges a profound and intimate desire to possess a vision of life and reality which brings together, connects with, transcends, and gives meaning to existence. It encompasses a dynamic personal exploration in three directions: it is introspective as we search for meaning; it looks outward as it searches for a connection; and it looks beyond reality as it searches for transcendence.”

The GES also created a questionnaire to identify spiritual needs, assessing the three dimensions of spirituality the figure most prominently in the literature: the intrapersonal, interpersonal, and transpersonal dimensions (Benito et al., 2014).

With regard to interventions, some studies have reported the benefits that a range of psychological interventions can have on the spiritual well-being of patients at the end of their lives (Gysels and Higginson, 2002; Newell, 2002; Uitterhoeve et al., 2004; Fegg, 2006; Lorenz et al., 2008; Price and Hotopf, 2009; Breitbart et al., 2010, 2012, 2015; Li et al., 2012; Houmann et al., 2014). In this field, counseling is one of the most widely used strategies (Gysels and Higginson, 2002; Newell, 2002; Edwin and Cassem, 2009; Porche et al., 2014), with benefits being reported for interventions within this framework in spiritual well-being for patients at the end of their lives (Rudilla et al., 2015). Meaning-centered psychotherapy has also been demonstrated to be effective in improving patients’ quality of life, reducing depression and despair, and encouraging spirituality (Breitbart et al., 2010, 2012, 2015). Cognitive–existential group psychotherapy has performed well in carers of palliative care patients (Kissane et al., 2003, 2006). Positive results have also been achieved using CALM (Managing Cancer and Living Meaningfully), as reported by Li et al. (2012): a reduction in the duration of depressive symptoms and death-related anxiety and an increase in patients’ spiritual well-being.

Two of the most interesting studies on specific interventions focused on the spiritual needs of family caregivers at the end of their lives are those carried out by Ando et al., 2007, 2010). Their research shows that clear benefits can be obtained by assisting patients in meaningfully reviewing their lives.

However, a systematic review of interventions regarding spirituality in palliative care patients (Rudilla et al., 2018) found there still to be no consensus on the method or protocol to follow for specific interventions. There are many reports in the literature of improvements in patients’ spiritual well-being, but these results are typically by-products rather than the main objectives of such interventions, with the focus being elsewhere. Such studies concern questions such as the meaning of patients’ lives, dignity, interpersonal relationships, and even issues related to patients’ emotional symptoms (such as anxiety and depression). Thus, studies such as that by Bernard et al. (2017) demonstrate that spirituality-focused interventions continue to be difficult to undertake, partly due to the way in which the issue is approached by professionals, which may be influenced by their own ideas and experience of what spirituality is. Therefore, the question remains as to how to tackle the issue of patient spirituality effectively without causing unnecessary distress or discomfort or dismissal of the topic.

Given the importance of spirituality for patients’ emotional well-being and the lack of protocols for systematic interventions in this respect reported in the literature, we designed a therapeutic interview for palliative care patients that focused on spirituality, named Kibo (“hope” in Japanese). This therapeutic interview consists of a brief introduction to facilitate a discussion of spirituality in a natural way, followed by the main questions, which seek to explore the three core dimensions of spirituality (the intrapersonal, interpersonal, and transpersonal). The interview protocol also contains a series of additional questions that seek to elicit introspection in the patient. The interview closes with some brief feedback from the health care professional to the patient, based on the answers provided during the interview.

### Aims

The main aim of this study was to evaluate the benefits that the Kibo therapeutic interview in palliative care patients can have for spirituality (across its three dimensions), demoralization, and resilience.

The assumption was made that the benefits of the interview would vary depending on the severity of each patient’s symptoms.

We proposed to test the following hypotheses:

Hypothesis 1. Participating in the Kibo intervention will be related to an improvement in spirituality.

Hypothesis 2. An improvement in spirituality will be related to improvements in resilience and demoralization.

Hypothesis 3. Patient symptoms will be related to the target variables.

## Materials and Methods

### The Development of the Kibo Intervention

The Kibo therapeutic interview was developed on the basis of the GES questionnaire and its underlying model of spirituality (Benito et al., 2014). Within this framework, a four-step intervention was developed:

#### Introduction

First of all, a brief introduction to the intervention is presented to the patient.

Next, the patient is asked questions regarding the three dimensions of spirituality:Intrapersonal spirituality. This concerns the relationship with oneself, satisfaction, a sense of achievement, a loss of autonomy, dependency, and failure.

(1) Interpersonal spirituality. This focuses on the issues of harmony, relationships with others, feeling loved, feeling at peace with others, and social isolation.
(2) Transpersonal spirituality. This last dimension comprises aspects such as awareness of transcendence, confidence, hope, legacy, connection with a greater reality, hopelessness, meaning in life, and powerlessness.

#### Closing Question

This consists of an open question that gives the patient the opportunity to freely express anything that he/she wishes.

#### Feedback

At the end of the intervention, the clinician provides the patient with a brief summary of the most meaningful data collected during the interview.

Questions for the different steps are shown in Table 1.

**TABLE 1:** The Kibo therapeutic interview.

Steps	Questions
(1) Introduction	0. I would like to get to know you better. For me, it is very important to know about the kind of person you are, what things bother you, and what things help you. Sometimes it is very difficult for us to explain how we feel and what we think. If you agree, we are going to spend some time talking about these things.
(2) Intrapersonal dimension	1. Tell me about yourself, your family, your childhood, the jobs you’ve had, and so on. What would you say have been important moments in your life? 1a. What do you think you are good at or would you consider to be your skills or strengths? 1b. Who are the most important people in your life? **1c. What are the things that you have most liked or enjoyed in your life?** 2. What are you most proud of? 2a. Is there anything that you would have liked to have done differently? 2b. Why is this so important for you? 3. **Right now: what makes you the most happy or gives you the most satisfaction or peace?** **What things come to mind?** **3.a. How do you feel at this point in your life?**
(3) Interpersonal dimension	4. What do you feel when you think of your loved ones? 4a. Is there anything that needs to be forgiven or fixed, anything that hasn’t been done? 4b. If you could do anything in this respect, what do you think you could do? 5. Is there anything that you would like to say or even repeat to your loved ones? Perhaps something that you feel is important to say. **6. What would you like to communicate to your loved ones?** **Any dreams or hopes that you may have?**
(4) Transpersonal dimension	7. If you had to tell me what kind of person you were, what would you say? 7a. What do you do in your day-to-day life? 7b. Would you like to do anything specific? 8. Do you have faith? Are you a believer? 8ai. How would you describe your relationship with God? 8bi. What link do you see between God and your illness? **8aii. What values or beliefs help you?** 8bii. What would you say your philosophy of life is? 8cii. What influence has your illness had on this?
(5) Closing question	7. Is there anything you need or would like to say or express?
(6) Feedback	8. Is there anything you need or would like to say or express?

*Questions in bold are used for therapy feedback. Questions 8aii, 8bii, and 8cii can be used as supplementary questions for patients with manifest religious beliefs.*

The therapeutic framework of the intervention is counseling, while the basis of the procedure is framed in cognitive behavioral therapy. The authors chose a semi-structured interview model for several reasons. On the one hand, based on previous research regarding interventions in spirituality at the end of life (Ando et al., 2007, 2010; Breitbart, 2007; Breitbart et al., 2010, 2012), the semi-structured interview maintains the structure of the theoretical model on which it is based and ensures the exploration and evocation of elements of the three dimensions of spirituality. On the other hand, it gives a margin of freedom to adapt to a broad concept of spirituality, understood as an essential dimension of the human being (Macrae, 1995; Tanyi, 2002; Sinclair et al., 2006; Vachon et al., 2009; Visser et al., 2010) and which, therefore, can to a certain extent mean different things to different people. The interview allows for adaptation to the previous answers given by the patient and allows the patient himself/herself to reflect, formulate, and structure his/her answers. It is in this process of reflecting on and formulating his/her own responses, based on life experience, that the patient comes into contact with the essential elements that have nourished his/her spirituality throughout life. In addition, with the final feedback from the interviewer based on the patient’s responses, the patient becomes even more aware of the elements that have been most central to his/her life, including elements from each of the three dimensions of spirituality. It is this process of reconnecting with the essential elements of his/her life, evoking them, and bringing them to consciousness that contributes to the patient’s ability to reconstruct his/her biography with meaning or to reaffirm the meaning he/she finds in his/her own biography, which is therapeutic (Ando et al., 2007, 2010; Breitbart, 2007; Breitbart et al., 2010, 2012). At the same time, this exploration and evocation of essential moments allows the detection of possible elements that could be risk factors for possible complications, such as guilt, disconnection, regrets, etc., that may be of interest in terms of working on them in a therapeutic way and working them out in a more adaptive way for the patient.

### Design, Procedure, and Participants

A parallel randomized controlled trial of two groups was undertaken. After reviewing prior research on the effectiveness of psychological interventions in spirituality in palliative care patients, we found that a number of studies did not provide precise details of the intervention design and so do not provide data that enable the standardized effect size to be determined. In the studies where this can be determined (Edelman et al., 1999; Goodwin et al., 2002), values of around 0.50 are found for the effect size. At this point, it is worth bearing in mind that a sufficient number of subjects are required for appropriate statistical analysis to be carried out, enabling their values to be assumed to be normal, and that a sufficient number of observations need to be carried out for multivariate analysis to take place. Therefore, the size of the experimental and control groups was set at 32. Taking this information into account, the experimental and control group size was fixed to 32.

The eligibility criteria for participants were as follows.

Inclusion criteria:

(a) Being over 18 years of age.
(b) Being in an advanced or terminal disease phase (following WHO criteria).
(c) Having preserved cognitive capacity.
(d) Having signed the informed consent document and confidentiality agreement, within the framework of the principles of the Declaration of Helsinki.

Exclusion criteria:

(a) Estimated time of survival. Patients with a life expectancy of 2 weeks or less were not to be interviewed.

**FIGURE 1:**
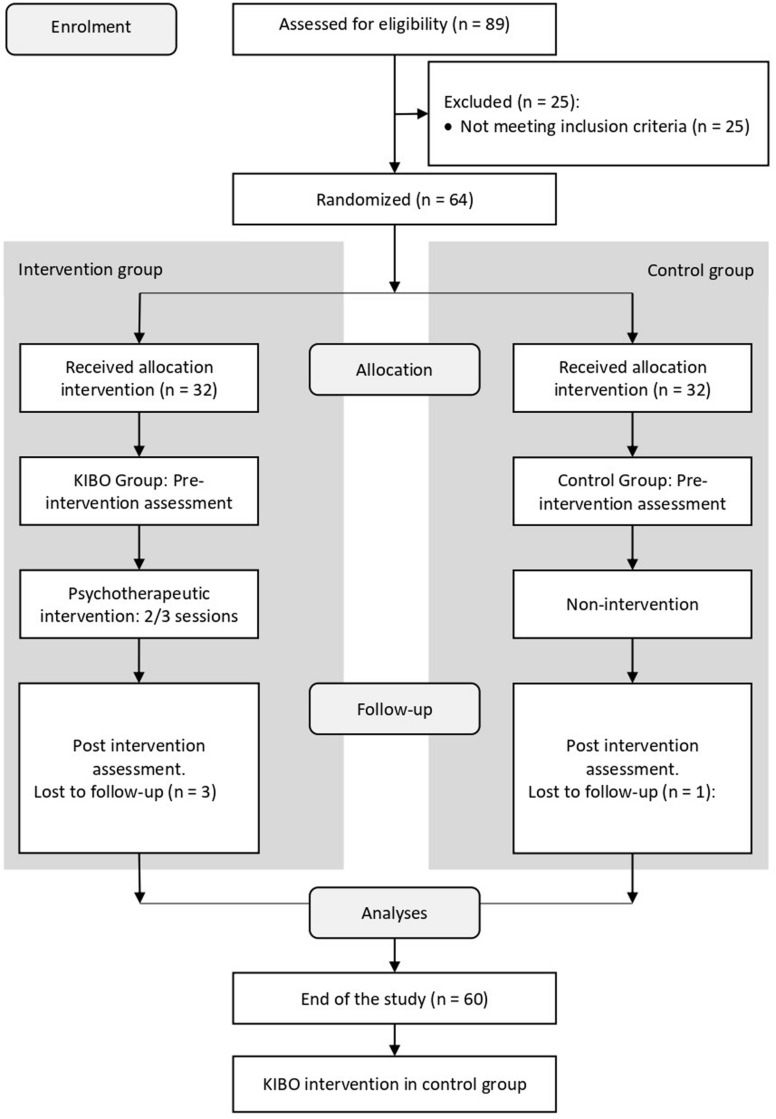
CONSORT Diagram.

Finally, 17 health professionals participated as interviewers, from 16 different health centers (see Appendix), and information on 60 palliative care patients during pre- and post-intervention time points was gathered (The CONSORT Diagram can be seen in Figure 1). The trial is properly registered at ClinicalTrials.gov (trial registry name: Intervention in Spirituality at the End of Life. The Kibo Protocol, URL: https://clinicaltrials.gov/ct2/show/NCT03995095, ClinicalTrials.gov registration number: NCT03995095). A description of the professionals and patients can be found in Table 2.

**TABLE 2:** Professionals’ and patients’ characteristics.

Professionals (*N* = 17)			Patients (*N* = 60)		
	
Profession	N	%	Gender	N	%
Psychologist	10	58.82	Women	30	50.00
Physician	5	29.42	Men	30	50.00
Nurse	1	5.88	Unit	**N**	**%**
Others	1	5.88	Palliative care unit	40	66.67
			Home care unit	20	33.33
			Marital status	**N**	**%**
			Single	7	11.67
			Married	41	68.33
			Divorced	2	3.33
			Widowed	8	13.33
			Unknown	2	3.33
			Age	**M**	**SD**
				65.18	11.66

Data were collected between March 2016 and March 2019. After checking for the inclusion criteria, participants were randomized assigned to the experimental and control groups. Once the groups were formed, participants went through three different stages:

(1) *Pre-intervention.* Participants answered the pre-intervention survey. It included sociodemographic data and several measurement instruments (see section “Outcome Measures” for the details).
(2) *Intervention.* In this second stage took place the intervention *per se*. Details of the intervention have been explained before and are shown in Table 1.
(3) *Post-intervention.* In this stage, the professional finished the intervention, giving the patient feedback based on the most meaningful answers collected through the interview.

In the end, the professional always gave the patient the opportunity to freely ask any questions and also to demand additional sessions. However, in the cases where additional sessions took place, these were not included in the present study.

Stages 1 and 3 lasted about 1 h. Stage 2, in which the intervention took place, lasted from 1 to 3 h, distributed in sessions of 1 h each in order to adapt to each patient’s needs and possibilities. Sessions of Stage 2 took place during the same week. Between each of the stages, a week went by. More specifically, between the end of the intervention and the post-intervention assessment, a week passed by.

### Research Ethics

The University of Valencia’s Ethical Committee on Human Research reported favorably on the study (H1447334931417), as it respects the principles of the Declaration of Helsinki, in the Council of Europe’s Convention on Human Rights, and complies with the requirements established in Spanish legislation in the field of biomedical research, personal data protection, and bioethics.

Control group participants received the regular help that the health care providers usually provide in these cases, plus the assessment of variables at two time points (equivalent to pre- and post-intervention assessment). It was the same for experimental group participants, with the only addition being the use of the Kibo therapeutic interview. The therapeutic interview was offered to the control group patients after the second moment of assessment (post-intervention), and it was implemented in those cases where the patient wanted it, although this was not always possible due to the nature of the medical condition of these patients.

Furthermore, all information about the study was provided to participants during the informed consent. No specific information on possible harms of the intervention was reported, because there is no precedent in the scientific literature pointing to harms of the interventions on which the Kibo interview is based. Patients were also informed that they could leave the study at any time, although no patient requested not to finish the study. The design of the interview allows the patient to evoke topics and to go into them in depth or not, according to his/her wishes. At the same time, at the end of the interview, there is an open-ended question in which the patient is allowed to freely express any issue that he/she wishes to discuss with the interviewer, giving rise to the detection of possible issues that may have been left pending, into which the patient wishes to go into more detail, or that he/she simply wishes to communicate to the interviewer. Finally, although the post-intervention measure is designed to be able to detect both improvements and possible worsening after the intervention, no further follow-up of the patient was carried out after the post-treatment assessment, mainly because the end-of-life characteristics of the participants made it difficult to carry out a subsequent follow-up over time.

### Outcome Measures

The research included the following measurement instruments:

(a) GES questionnaire (Benito et al., 2014). It is composed of six open questions designed to facilitate the trust and disclosure of the patient, obtain his/her biography, and learn about his/her inner world. These six open questions are followed by eight items that assess spirituality as a general factor with three spiritual dimensions: intrapersonal, interpersonal, and transpersonal. The patient responds to what extent he/she identifies with the items using a scale from 0 (“nothing”) to 4 (“much”). For participants in this study, the internal validity of this instrument is α = 0.843.
(b) Brief Resilient Coping Scale (Sinclair and Wallston, 2004) in its Spanish version (Tomás et al., 2012). It is a four-item scale where each item can be scored from 1 (“strongly agree”) to 5 (“totally disagree”). Higher scores reflect greater resilience. For participants in this study, the internal validity of this instrument is α = 0.889.
(c) The brief scale of demoralization (Galiana et al., 2017). It assesses the demoralization syndrome by means of five items comprising the five dimensions of the Demoralization Scale (Kissane et al., 2004): loss of meaning, helplessness, disheartenment, dysphoria, and sense of failure. Items are scored on a frequency Likert-type scale, from 0 (“never”) to 4 (“all the time”). For participants in this study, the internal validity of this instrument is α = 0.913.
(d) Edmonton Symptom Assessment System (ESAS) (Bruera, 1993) in its Spanish adaptation (Centeno et al., 2004). This list of 10 numerical scales assesses the intensity of symptoms in a specific period of time depending on the patient’s condition. Patients select the number that best indicates the intensity of each symptom. For participants in this study, the internal validity of this instrument is α = 0.774.

### Data Analyses

To study the effectiveness of the intervention, one multivariate analysis of covariance (MANCOVA) and three mixed univariate analyses of covariance (ANCOVAs) were performed. In each of them, the between-subjects independent variable was group, with two categories (control vs. intervention group), and the within-subjects independent variable was time, also with two categories (pre- and post-intervention). Scores on the ESAS measured before the intervention were used as a covariate to control for individual differences. The MANCOVA included as dependent variables the dimensions of spirituality: intra-, inter-, and transpersonal spirituality. Regarding the ANCOVAs, the first ANCOVA tested the effects of time, group, and their interaction on resilience. The second ANCOVA tested the effects of the independent variable and its interaction on symptom severity. Finally, the third ANCOVA studied the effect of the intervention on quality of life.

In all the cases, the aim was to evaluate the differences in the means of the dependent variables for the different categories of the independent variables, and also of the effect of the interaction, while controlling for the different levels of symptom severity. When studying the effect of an intervention, our goal is to detect differences produced by the interaction: the effect of time will indicate if the variables change as times go by, for all the patients, regardless of whether they have participated in the intervention or not; the effect of group, in turn, will indicate if the groups have different levels on the dependent variables, without taking into account the time point; finally, the intervention effect will be the one indicating if the effect of time is different for the two groups. We expect the interaction effect to be statistically significant, and to favor the intervention group, as it will mean that time has produced an effect in the patients participating in the intervention and a different one (smaller, or none at all) in those not participating in it.

Assumptions were tested to check for normality, linearity, univariate and multivariate outliers, and multicollinearity, with no serious violations noted. The effect size was assessed with partial eta-squared (η*^2^*), taking as cutoff criteria those proposed by Cohen (1992):0.02, 0.13, and 0.26, for small, medium, and big effects, respectively.

All analyses were carried out using the IBM SPSS Statistics software (version 24).

## Results

The MANCOVA assessed the effect of the variables time and group and their interaction on the dimensions of spirituality. After adjusting for ESAS, there was a statistically significant and big effect size of the interaction time × group [*F*(3,55) = 5.750, *p* = 0.002, η*^2^* = 0.239]. Neither of the main effects was statistically significant [time: *F*(3,55) = 0.648, *p* = 0.587, η*^2^* = 0.034; group: *F*(3,55) = 1.101, *p* = 0.357, η*^2^* = 0.057]. The covariate, ESAS, was non-significantly related to spirituality quantification: *F*(3,55) = 1.494, *p* = 0.226, η*^2^* = 0.075.

**FIGURE 2:**
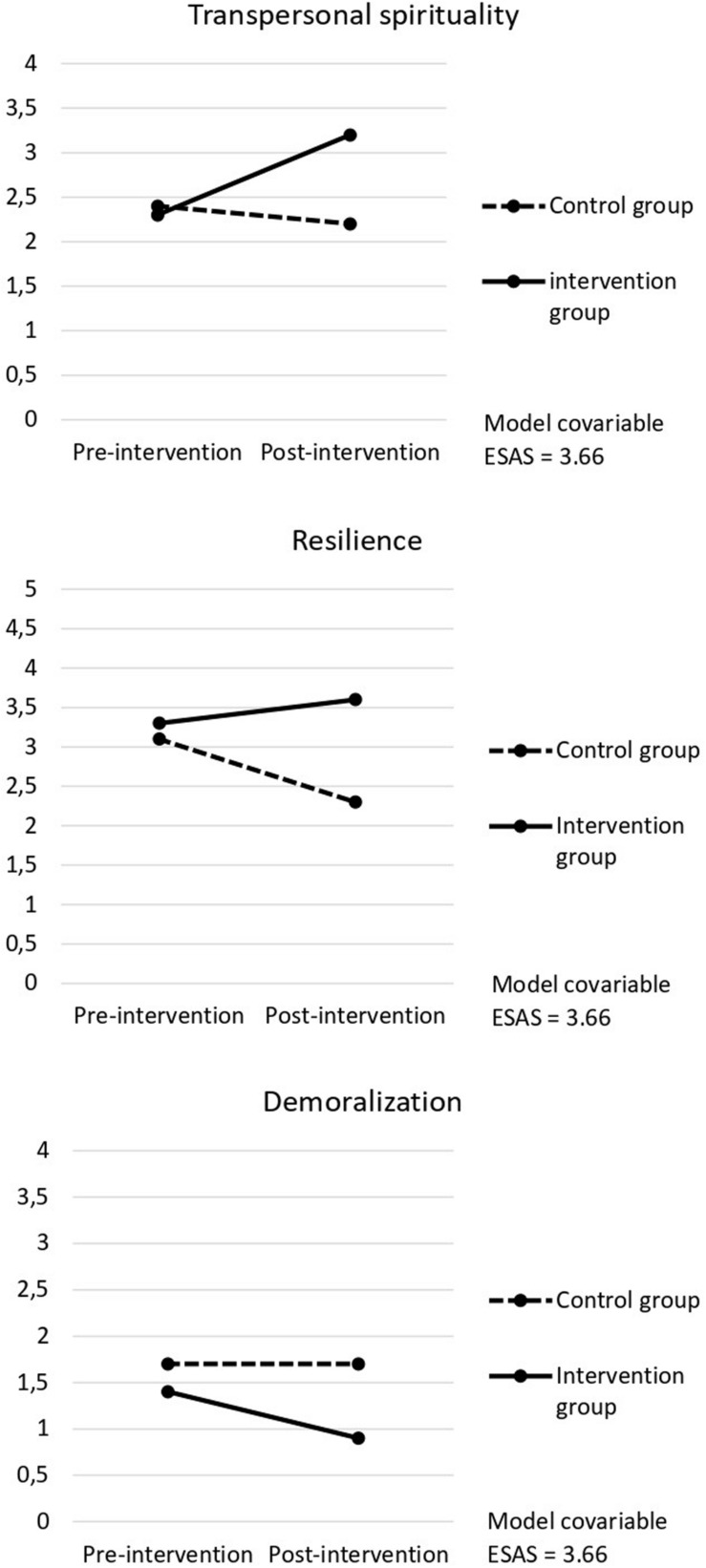
Estimated pre- and post-intervention marginal means for the intervention and control groups, for the variables with a statistically significant interaction effect.

The statistically significant effect of the interaction pointed to the effectiveness of the intervention to improve patients’ levels of spirituality. As shown in Table 3, follow-up ANOVAs pointed to a statistically significant effect of the intervention on the dimension of transpersonal spirituality. As can be seen in Figure 2, the effect favored the intervention group, with patients improving their levels of transpersonal spirituality over time, whereas patients of the control group showed a small decrease in the dimension. Details of the means can be seen in Table 4.

**TABLE 3:** Follow-up ANOVAs for the effects of the interaction on the dimensions of spirituality.

Spirituality dimensions	df_*effect*_	df_*error*_	*F*	*p*	η *^2^*
Intrapersonal spirituality	1	57	0.166	0.686	0.003
Interpersonal spirituality	1	57	1.371	0.247	0.023
Transpersonal spirituality	1	57	16.647	<0.001	0.226

**TABLE 4:** Pre- and post-intervention mean values for the intervention and control groups.

Variables	Intervention group		Control group	
		
	Pre-intervention	Post-intervention	Pre-intervention	Post-intervention
Intrapersonal spirituality	2.97	3.64	2.82	3.42
Interpersonal spirituality	3.34	4.37	3.29	3.46
Transpersonal spirituality	2.39	3.06	2.20	2.09
Resilience	3.43	3.71	2.85	2.58
Demoralization	1.23	0.87	1.88	1.76

The ANCOVA studying the effect of the intervention on resilience also pointed to its effectiveness. Specifically, the effects of time [*F*(1,57) = 6.518, *p* = 0.013, η*^2^* = 0.103), group [*F*(1,57) = 5.864, *p* = 0.019, η*^2^* = 0.093), and the interaction time^∗^group [*F*(1,57) = 12.591, *p* = 0.001, η*^2^* = 0.181] were statistically significant. Also, the covariate was significantly related to spirituality quantification: *F*(1,57) = 32.155, *p* < 0.001, η*^2^* = 0.361.

These results point to the effectiveness of the intervention for improving patients’ resilience by an important amount. As it happened with transpersonal spirituality and is graphically presented in Figure 2, the effect favored the intervention group, with patients improving their levels of resilience after the intervention, whereas patients of the control group showed a small decrease in their mean. Details of the means can be seen in Table 4.

Finally, when demoralization was studied, again, the effects of time [*F*(1,57) = 4.673, *p* = 0.035, η*^2^* = 0.076], group [*F*(1,57) = 5,706, *p* = 0.020, η*^2^* = 0.091], and the interaction time × group [*F*(1,57) = 4.327, *p* = 0.042, η*^2^* = 0.071) resulted as statistically significant, and also, the ESAS was significantly related to spirituality quantification [*F*(1,57) = 22.479, *p* < 0.001, η*^2^* = 0.283].

When the means of demoralization were examined, a higher decrease in the levels of demoralization for patients in the intervention group was observed, when compared to patients in the control group (see Table 4). This decrease is graphically shown in Figure 2.

## Discussion and Conclusion

Several studies (Baines and Norlander, 2000; Chochinov, 2002; Draper, 2012; Otis-Green et al., 2012; Puchalski, 2012), as well as the responsible entities in the field (World Health Organization, 2002), point out the importance of attending to the spiritual needs of patients at the end of their lives. Attention to these needs concerns not only the spiritual dimension of each person but also the other human spheres with which the person is related, as spirituality has been seen to be related to the physical and psychological well-being of people (Koenig et al., 2004; Delgado-Guay et al., 2011; Hui et al., 2011; Kandasamy et al., 2011; Rajmohan and Kumar, 2013; Haugan, 2014; Fombuena et al., 2015). Attending to these needs has enormous potential to reduce the suffering of these patients at different levels, from better control of symptoms (Fombuena et al., 2015) to a greater acceptance of the end-of-life situation and fewer concerns about death (Edmondson et al., 2008; Corr and Corr, 2012).

Despite the extensive literature in this regard, we have observed that very few studies propose a protocol to address these needs, and those that do often present no data on its effectiveness (Miller and Thoresen, 2003; Chochinov and Cann, 2006; Selman et al., 2011; Galiana et al., 2013). Therefore, it is essential to provide data in this sense that allow an integrated proposal of intervention, which takes into account the most important basic elements to date and is based on empirical data, as well as providing data on its effectiveness and even on its relationship with other variables that are also fundamental to the welfare of human beings, such as resilience, or those that are so closely related to the suffering of these patients, such as demoralization.

Thus, the reasons for developing the Kibo intervention were: (1) the need to address spiritual needs in patients at the end of life, (2) the lack of consensus and protocol for spirituality-specific interventions, and (3) the questions of tackling spirituality issues with patients in an effective and respectful way. We hope to have contributed to helping solve these problems for the following reasons:

- The proposed protocol is based on scientific literature, taking into consideration those elements that have shown greater effectiveness in addressing the spiritual needs of patients at the end of life (Ando et al., 2007, 2010; Breitbart, 2007; Breitbart et al., 2010, 2012). At the same time, the theoretical model in which it is framed, the GES model of spirituality (Benito et al., 2014), has been constructed empirically. We consider that all of this makes the Kibo intervention proposal a solid one that gathers the most essential elements of previous research in this field.
- On the other hand, and always taking into account previous research, we have tried to address the issue of spirituality in a way that is not only effective but also respectful. At the same time, the theoretical model in which the intervention is framed contemplates a broad definition of spirituality, encompassing elements supported by scientific literature that is not restricted solely to aspects related to religious beliefs. To ensure that this broad definition of spirituality is addressed, the interview proposes different questions to be asked, depending on the previous answers given by the patient, thus adjusting to the individual profile of what spirituality means for that particular person (for example, different groups of questions to be used depending on whether the person is a believer or not). Despite keeping these questions optional to ensure that a broader definition of spirituality (as an essential dimension inherent to every human being) is addressed, the Kibo interview maintains at the same time the general three-dimensional structure based on the empirically constructed GES model.

We present a therapeutic interview that draws from previous works in this field, in the hope of contributing to the attention to spiritual needs in the context of the end of life, specifically in palliative care patients. Our findings point to this interview as an effective way of increasing spirituality in patients at the end of life, facilitating the attention to their spiritual needs. Based on the significant results, the effect size measures revealed the quality of the intervention in terms of approximately 24% of differences accounted for by spirituality, focused on the transpersonal dimension.

Our results also support the effect of the therapeutic interview in reducing demoralization and increasing resilience in palliative care patients. This finding is also in line with previous research (Fombuena et al., 2015). Based on the significant results, the effect size measures revealed the quality of the intervention, approximately 18.1% for resilience and 7.1% for demoralization. This finding is particularly meaningful since a relationship between symptoms, resilience, and demoralization was found. However, no similar relationship was found for spirituality.

Finally, our findings are consistent with the view that even though spirituality, resilience, and demoralization are related, the concept of spirituality is wider than those of resilience and demoralization and, therefore, that there is a need to evaluate spirituality and carry out interventions in this regard.

### Limitations and Future Research

A limitation of this study is that, despite presenting a structured intervention, there are numerous elements of it that are not standardized. Although this makes sense for the sake of greater adaptation to the variability of the concept of spirituality for different people, it is true that it presents the limitation that in terms of its psychometric properties, this lack of standardization is a strong limitation. However, the standardization of the reliability and validity of the intervention protocol is reported with the mean variations between the intervention and control groups in key reliable measures that provided evidence for its validity (spirituality, resilience, coping, demoralization), through specific recording and mean variation between the initial and final variations in each group.

On the other hand, another limitation of this study is that the effects of the intervention could not be measured in a longitudinal way, but only at a single moment after the intervention. In this sense, the design includes a randomized control group, and the statistically significant and large effect size of the interaction time × group [*F*(3,55) = 5.750, *p* = 0.002, η*^2^* = 0.239] supported the effect across time in a short period. Nevertheless, not including a follow-up is a methodological limitation in seeing the sustainability of the effects, but in this applied research field, it is somehow difficult to include due to the prognosis of end-of-life participants.

Finally, even though pre- and post-intervention questionnaires were filled either by the patients or with a research assistant other than the professional applying the Kibo protocol another limitation of the study is not including additional blind raters from among other professionals attending to the patients about the benefits of the intervention, in order to avoid the possible influence of the interviewer on the pre- and post-intervention data, which would have enriched the study.

Future research could focus on the effect of the Kibo therapeutic interview on a broader sample, taking into consideration other variables such as gender, health care setting (domiciliary, hospice, etc.) and main diagnosis [such as Chronic Obstructive Pulmonary Disease (COPD), Amyotrophic Lateral Sclerosis (ALS), etc.] and cultural context, and also its effect on vulnerable populations in whom the end-of-life stage is harder to determine but who also present intense spiritual needs. It would also be interesting to analyze the effects of this interview on family caregivers, mourners, and health care professionals, especially palliative care staff. Also, a qualitative analysis of the information provided by patients could be of great interest, complementing the results found here.

### Practical Implications

The therapeutic interview that we propose can be carried out by health professionals of different fields and specialization, since its characteristics allow it to be adapted to the individual features of the interviewer while remaining organized at the same time. It is flexible but well structured and well defined.

At the same time, the therapeutic interview presented in this work has some features that make it particularly useful in working with the patient’s emotions. The interview does not only take into consideration the cognitive aspects of spirituality; it also assesses the emotions and way of feeling of the patients. Many of its questions also include the explicit assessment of feelings regarding what is being brought into the conversation. We believe that this is an important part of the structure of the interview, making it easier for the interviewer to make sure that emotions are also expressed and talked about.

We believe that the Kibo therapeutic interview is a useful means to attend to the spiritual needs of patients at the end of life, and time and further empirical data will help to elucidate with greater depth its full potential in this context.

## Data Availability Statement

The datasets generated for this study are available on request to the corresponding author.

## Ethics Statement

The studies involving human participants were reviewed and approved by Comité de Ética en Investigación en Seres Humanos University of Valencia N° H1447334931417. The patients/participants provided their written informed consent to participate in this study.

## Author Contributions

All authors made a substantial contribution to the concept or design of the work; or acquisition, analysis or interpretation of data; drafted the article or revised it critically for important intellectual content; approved the version to be published; and have participated sufficiently in the work to take public responsibility for appropriate portions of the content.

## Conflict of Interest

DR was employed by company Air Liquide Healthcare. The remaining authors declare that the research was conducted in the absence of any commercial or financial relationships that could be construed as a potential conflict of interest.

## Appendix

Participating centers.

Hospital Dr. Moliner.

Consorcio Hospital General Universitario de Valencia.

Fundación Jiménez Díaz.

Hospital San José Salud, Aragón.

Hospital San José Salud, Aragón.

Hospital La Magdalena, Castellón.

Hospital Dr. Moliner.

Instituto Valenciano De Oncología (IVO).

Hospital de Denia.

Hospital Clínico de Valencia.

Médico Adjunto del Área Médica integral del Hospital Pare Jofre.

Centro Sociosanitario Fundación Santa Susana.

Hospital Dr. Peset. Valencia.

Centro de cuidados de La Laguna.

Hospital Universitario y Politécnico La Fe.

Asociación Carena.

Hospital Provincial de Castellón.
